# Supplementary material for: Processing of motion boundary orientation in macaque V2
Source: eLife. 2021 Mar 24;10:e61317. doi: 10.7554/eLife.61317 (PMC8026216; doi:10.7554/eLife.61317)
Supplement: Supplementary file 1. [file elife-61317-supp1.docx]

**Number of V2 electrodes (and neurons) from which MB neurons were recorded.**

|  | Right hemisphere | Left hemisphere |
| --- | --- | --- |
| Monkey S | 10 (43 neurons) | 9 (13 neurons) |
| Monkey W | 1 (1 neuron) | 7 (13 neurons) |
