# Supplementary material for: Processing of motion boundary orientation in macaque V2
Source: eLife. 2021 Mar 24;10:e61317. doi: 10.7554/eLife.61317 (PMC8026216; doi:10.7554/eLife.61317)
Supplement: Supplementary file 2. — Note: The right hemisphere array in monkey W had a low electrode number since two thirds of its electrodes were located in V1. [file elife-61317-supp2.docx]

**Number of V2 electrodes (and neurons) from which CP was calculated.**

|  | Right hemisphere | | Left hemisphere | |
| --- | --- | --- | --- | --- |
|  | Coherence | Brightness | Coherence | Brightness |
| Monkey S | 5 (7 neurons) | 6 (17 neurons) | 5 (5 neurons) | Not tested |
| Monkey W | 0 | Not tested | 3 (3 neurons) | Not tested |

Note: The right-hemisphere array in monkey W had a low electrode number since two thirds of its electrodes were located in V1.
